# A Highly Reproducible Micro U‐Well Array Plate Facilitating High‐Throughput Tumor Spheroid Culture and Drug Assessment

**DOI:** 10.1002/gch2.202000056

**Published:** 2020-11-04

**Authors:** Kuang‐Wei Wu, Ching‐Te Kuo, Ting‐Yuan Tu

**Affiliations:** ^1^ Department of Biomedical Engineering National Cheng Kung University Tainan 70101 Taiwan; ^2^ Department of Mechanical and Electro‐Mechanical Engineering National Sun Yat‐sen University Kaohsiung 80400 Taiwan; ^3^ Medical Device Innovation Center National Cheng Kung University Tainan 70101 Taiwan; ^4^ International Center for Wound Repair and Regeneration National Cheng Kung University Tainan 70101 Taiwan

**Keywords:** cisplatin, CO_2_ laser, microwells, multicellular tumor spheroids, permeability coefficient, tumor models

## Abstract

3D multicellular tumor spheroids (MCTSs) have recently emerged as a landmark for cancer research due to their inherent traits that are physiologically relevant to primary tumor microenvironments. A facile approach–laser‐ablated micro U‐wells–has been widely adopted in the past decade. However, the differentiation of microwell uniformities and the construction of arrays have all remained elusive. Herein, an improved laser‐ablated microwell array technique is proposed that can not only achieve arrayed MCTSs with identical sizes but can also perform high‐throughput drug assessments in situ. Three critical laser ablation parameters, including frequency, duty cycle, and pulse number, are investigated to generate microwells flexibly with a range from 170 to 400 μm. The choice of microwells is optimally arranged into an array via precise control of horizontal spacing (*d*_x_) and vertical spacing (*d*_y_) amenable of cell‐loss‐free culture during cell seeding. Harvested T24, A549 and Huh‐7 MCTSs from the microwell array correspond to approximately 75 to 140 μm in diameter. Anticancer drug screening of cisplatin validated IC50 values in 2D and MCTS conditions are 3.5 versus 9.1 μM (T24), 11.8 versus 277.7 μM (A549) and 33.5 versus 52.8 μM (Huh‐7), and the permeability is measured to range from 0.042 to 0.58 μm min^−1^.

## Graphical abstract

A facile and highly reproducible laser‐ablated micro U‐well with optimal array arrangement is demonstrated. Cell‐loss free formation of multicellular tumor spheroids is performed in 96‐well plates for high‐throughput drug screening of cisplatin. Dose‐response assays are investigated on T24, A549, and Huh‐7 in both 2D and MCTS conditions. The permeability dynamics of each type of MCTS are examined.

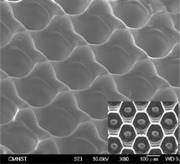

## 1 Introduction

Cancer has remained one of the leading causes of mortality worldwide each year.^[^ ^1^ ^]^ In vitro tumor models have shown great promise for anticancer drug development and provided working insights into tumor growth, proliferation, angiogenesis, and drug delivery. Among them, multicellular tumor spheroids (MCTSs) have surfaced as one of the most commonly used models for recapitulating several crucial elements of tumors.^[^ ^2^ ^]^ The MCTS is a highly complex 3D arrangement of cells that provides researchers with a physiologically and pathologically relevant microenvironment to study tumor biological functions. The cell‐cell and cell‐extracellular matrix (ECM) interactions of cells in the MCTS constitute gradients of nutrients, gas exchange, metabolic waste, cell proliferation, and drug access and mimic the structure and signaling of early‐stage avascular tumors.^[^ ^3^ ^]^

Microwell technology produced by microfabrication has emerged as a revolutionary tool over the past decade because it provides structurally confined microstructures for the highly consistent and reproducible growth of MCTSs.^[^ ^4^ ^]^ In recent research, cell‐loss‐free microwell arrays were designed in either honeycomb arrangements^[^ ^5^ ^]^ or inverted‐pyramidal opening cylindrical microwell structures,^[^ ^6^ ^]^ in addition to others.^[^ ^7^, ^8^ ^]^ These studies shared similar concepts of minimizing the spacing between microwells to avoid undesired cell settling as well as to prevent cell loss from excessive washing steps, and they were able to greatly enhance the production of harvestable MCTSs within a defined area. However, these methods require either conventional lithographic processes in cleanroom settings or the creation of master molds with added replica molding procedures, unavoidably increasing the cost and time consumption.

The application of CO_2_ laser ablation in microfabrication has offered a remarkably economical and rapid alternative for generating 3D MCTSs using size‐controlled microwells.^[^ ^9^, ^10^, ^11^, ^12^ ^]^ We previously demonstrated that applying CO_2_ laser ablation on a conventional polystyrene (PS) petri dish/microtiter plate could result in a U‐shaped microwell array, termed micro U‐well, that facilitated the formation of size‐controlled MCTSs and is suitable for simple in‐house rapid prototyping.^[^ ^10^, ^12^ ^]^ However, other studies using different laser systems have shown varying PS microwell geometries after laser processing.^[^ ^13^, ^14^ ^]^ Additionally, due to the thermal manufacturing of CO_2_ laser ablation, the heat‐affected recast region of the microwells has not been optimized for cell‐loss free operation as well as the additional steps inevitably involved during the cell removal step after the initial cell seeding. These notions prompt a call for laser parameter unification and the search for an optimal cell‐loss free microwell arrangement.

Laser‐ablated microwell‐produced MCTSs have had tremendous applications, including studies on chemotherapeutic assessment,^[^ ^15^ ^]^ photothermal treatment,^[^ ^12^ ^]^ and the inhibition of the epithelial‐mesenchymal transition.^[^ ^16^ ^]^ The efficiency of penetration of therapeutic agents through multilayer tumors depends strongly on the permeability across biological membranes.^[^ ^17^ ^]^ However, permeability dynamics in MCTSs have only been investigated in a limited capacity,^[^ ^18^, ^19^ ^]^ and its effect on the drug susceptibility on different types has remained elusive. Therefore, developing a quantification method to assess the permeability in MCTSs may provide a window for the better understanding of anticancer drug evaluation.^[^ ^20^ ^]^

Here, we aimed to develop a highly reproducible laser‐ablated micro U‐well array with an optimal arrangement through a CO_2_ laser system for the formation of MCTSs from various cell types suitable for the screening of drugs and quantification of the MCTS permeability coefficient (*P*_MCTS_). The geometric dimensions of the microwells were characterized according to the laser command parameters of frequency, number of pulses, and duty cycle. The microwell array was optimally arranged to minimize cell loss during the seeding process and to enhance the size uniformity of MCTSs. The formation of MCTSs and anticancer drug screening using cisplatin were validated in various cancer cells, and the quantification of the *P*_MCTS_ in these MCTSs was proposed.

## 2 Results and Discussion

### 2.1 Characterization of Microwells

**Figure 1:**
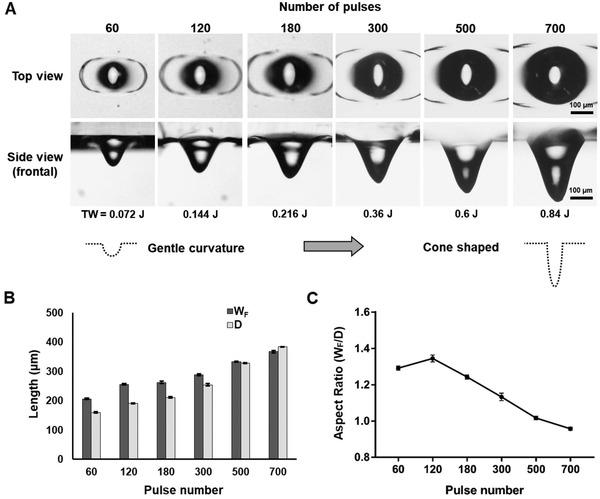
Microwells fabricated via various numbers of pulses to generate a wide range of sizes. A) Demonstration of gradually enlarged microwell structures at pulse numbers from 60 to 700 at 5 kHz and a 60% duty cycle. B) Characterization of microwell frontal widths (*W*_F_) and depths (*D*). C) Aspect ratio of microwells (aspect ratio = *W*_F_/*D*). All error bars represent the standard deviation of the measurements (*n* = 5).

The first set of experiments was to understand the role of the pulse number with respect to the morphology of the microwell (**Figure** **1**). Since the power was directly proportional to the pulses generated in each ablation, the microwell structures were investigated by fixing the frequency at 5 kHz and duty cycle at 60%, with pulses ranging from 60 to 700. Representative top and side views of the microwells delineated the morphological features under bright field microscopy (Figure 1A). In the top view, the wing‐like structure of the recast region could be clearly observed, with its size increasing in proportion to the increased pulses. The dark region of an “eye” structure in the center of the microwell represented the light deflected by the curved geometry of the microwell surface, and the bright spot indicated a flat bottom. This result may be explained by that the laser beam is slightly elliptical in shape,^[^ ^21^ ^]^ which in turn induced an asymmetrical recast pattern along the horizontal direction. In the side view, the microwells displayed a more conical shape with an increasing number of pulses. Gradual increases in *W*_F_ and *D*, as defined in the Section 4, were recorded, suggesting that under the power levels ranged from 0.072 to 0.84 J, the *W*_F_ ranged from 200 to 350 μm, and the *D* ranged from 150 to 360 μm (Figure 1B and Table S1, Supporting Information). The aspect ratio (AR) was defined as *W*_F_/*D*, which was maintained at ≈1.3 at 60 and 120 pulses and then gradually dropped to ≈1 at 700 pulses (Figure 1C). The results of correlated works and microwell sizes are generally in agreement with the trend shown in the previous findings reported by us^[^ ^10^, ^12^ ^]^ and others.^[^ ^13^, ^14^ ^]^ However, the varying microwell geometries reported from different studies suggest further investigations on a wider range of laser parameters maybe crucial.

**Figure 2:**
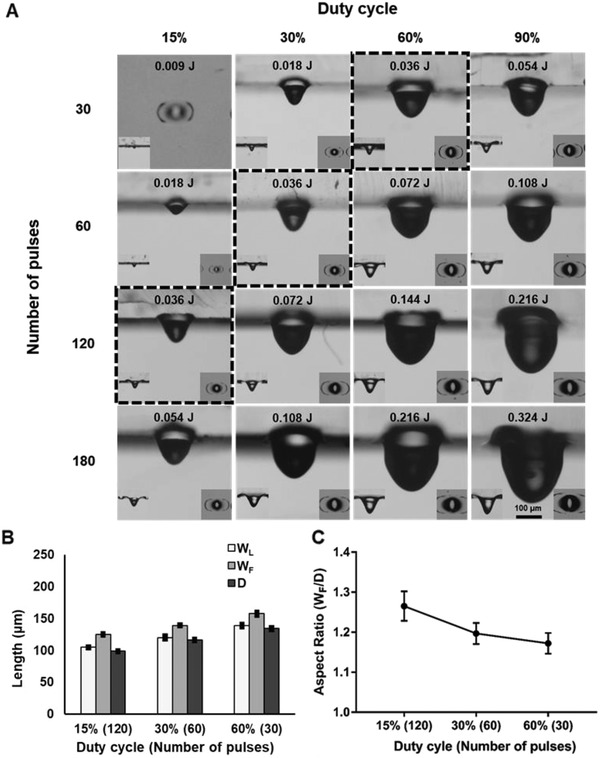
Microwells fabricated via a combinations of a range of duty cycles and number of pulses. A) Representative views of microwell features with respect to a range of pulse numbers from 30 to 180 at laser powers ranging from 1.5 to 9 W, where the black dotted lines indicate the microwells fabricated under the same total work (TW) highlighted on top of each image. B) Characterization of frontal and lateral widths and depths of microwells, and C) aspect ratio of microwells, highlighted by the black dotted lines in (A). All error bars represent the standard deviation of the measurements (*n* = 5).

Therefore, the effects of the duty cycle at 15%, 30%, 60%, and 90% (**Figure** **2**) and the frequency at 1, 5, 10, and 20 kHz were subsequently investigated (Figure S1, Supporting Information). At fixed pulses of 30, 60, 120, and 180, the dimensions of the microwell (both width and depth) increased as the duty cycle increased from 15% to 90%. (Figure 2A). The lateral side view of the microwell, compared to the frontal conical geometry, exhibited a more “bullet‐like” shape. As defined by Equation (1), under a fixed total work (TW) (highlighted by the different colored dotted lines), the microwells presented similarities in size and shape. The size of the microwells measured at the same TW showed that the duty cycle could result in slight increases in the *W*_F_ from 125 to 160 µm, in the lateral width (*W*_L_) from 100 to 140 µm and in the *D* from 100 to 135 µm (Figure 2B), corresponding to the AR dropping slightly from 1.27 to 1.17 (Figure 2C and Table S2, Supporting Information). To further elucidate the effect of the frequency, the laser command signal was tested at a fixed TW. Under a fixed TW of 0.108 J, the dimensions of the two side views of the microwell structures increased with increasing frequency from 1, 5, 10, and 20 kHz pulses at 36, 180, 360, and 720, respectively (Figure S1A, Supporting Information). At higher frequencies, the dimensions of the microwells were distinctly increased as follows: for *W*_L_, from 155 to 380 µm; for *W*_F_, 190 to 380 µm; and for *D*, from 150 to 390 µm (Figure S1B, Supporting Information). The AR decreased drastically from 1.26 to 0.96 (Figure S1C and Table S3, Supporting Information). Under different combinations of pulses, frequencies, and duty cycles, the results indicated that the microwell maintained its overall contour in both frontal and lateral geometries, and gradually increased in dimensions as TW enhanced. In addition, the microwell fabrication presented a highly reproducible and consistent manner that suggested varying microwell geometries shown in the previous studies could be attributed to other reasons in addition to the laser parameters.^[^ ^13^, ^14^ ^]^ Considering that different sizes and shapes of microwells could be fabricated upon using different laser pulse parameter, the conditions of 5 kHz, 60% duty cycle and 180 pulses were selected for further investigation on the optimal microwell arrangement and the growth of MCTSs.

Since the industrial CO_2_ laser was first demonstrated as an effective alternative for the rapid microfabrication of microfluidic systems in 2002,^[^ ^22^ ^]^ abundant research has presented various biomedical applications of CO_2_ laser‐ablated lab‐on‐a‐chip devices.^[^ ^23^, ^24^, ^25^, ^26^ ^]^ Laser‐ablated microwells using polyester were first demonstrated by ﻿Selimovi et al. in 2011;^[^ ^9^ ^]^ those using PS were demonstrated by our group in 2014;^[^ ^10^ ^]^ and those using polydimethylsiloxane (PDMS) were demonstrated by Albritton et al. in 2016.^[^ ^11^ ^]^ Application of these platforms to multicellular cell spheroid culture has resulted in significant advances in many fields of tissue engineering,^[^ ^27^ ^]^ such as stem cell therapy,^[^ ^28^ ^]^ neurological disease,^[^ ^29^ ^]^ and cancer research.^[^ ^10^, ^16^ ^]^ Among these materials, we previously showed that the concave and biocompatible natures of the PS microwells can be in‐house fabricated and easily accessible for most biological and medical laboratories.^[^ ^10^, ^12^ ^]^ However, the design of most conventional laser cutter systems confined the laser beam to travel through several reflective mirrors and a moving laser head prior to ablation. These movable parts required constant cleaning and may have resulted in variation in the laser incidence angle and the beam energy that subsequently affected the morphology of the microwells obtained, as shown in our^[^ ^12^ ^]^ and other previous studies.^[^ ^13^, ^30^ ^]^ Here, we presented a lab‐built CO_2_ laser system with a stationary laser head to study the effects of parameters on the laser ablation process. Through a reductionist approach, the laser commands of pulses (Figure 1), duty cycle (Figure 2), and frequency (Figure S1, Supporting Information) were independently investigated with the corresponding TW quantified for each ablation. For the first time, microwell structures can be consistently reproduced, and the geometric distributions were scrutinized using the *W*_F_, *W*_L_, *D*, and AR, which is believed to provide in‐depth guidelines on the choice of parameters.

### 2.2 Optimization of Microwell Arrangement and the Morphology of Multicellular Tumor Spheroids

**Figure 3:**
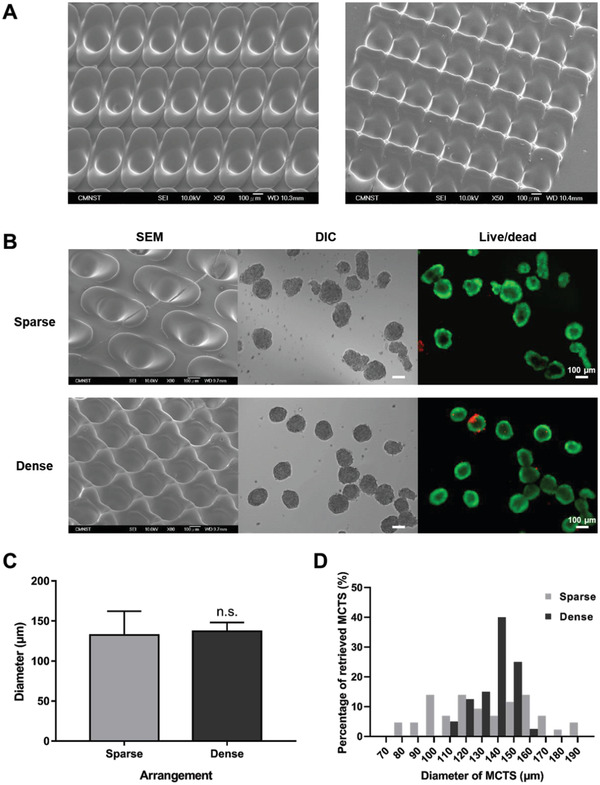
Comparison of Huh‐7 MCTSs formed in sparse and dense microwell array arrangements. A) Scanning electron microscopy (SEM) images of different microwell arrangements showing the improved progress of arrangements. B) SEM images of the microwell array, differential interference contrast (DIC) and fluorescent (live/dead staining) images showing the morphology of Huh‐7 MCTSs formed in sparse (*d*_x_ = 640 µm; *d*_y_ = 320 µm) and dense (*d*_x_ = 460 µm; *d*_y_ = 200 µm) microwell array arrangements. C) The diameters of the MCTSs retrieved from the sparse and dense microwell array arrangements (*n* = 50). D) Frequency distribution of Huh‐7 MCTS sizes in sparse and dense microwell array arrangements.

We further investigated the arrangement of microwells in different spacing conditions, and the corresponding formation of MCTSs in sparse/dense microwell arrangement designs were compared (**Figure** **3**). Several microwell spacing conditions were tested, in which two types of parallel arrangements, as shown in scanning electron microscopy (SEM) images, were worth noting (Figure 3A). First, the laser‐ablated microwells showed highly reproducible, smooth, and debris‐free geometry in both concave microwell and the wing recast region. Each of the microwells could be consistently fabricated without affecting one another (left, Figure 3A). Interestingly, if the recast regions were overlapped through reducing the space between microwells, the recast region was found to form a wall in between the microwells, and the microwell presented a “honeycomb”‐like shape (right, Figure 3A). To compare the effects of different microwell arrangements, two spacing conditions were investigated: Sparse (*d*_x_ = 640 µm; *d*_y_ = 320 µm) and dense (*d*_x_ = 460 µm; *d*_y_ = 200 µm), as defined in Section 4. The conditions used for the sparse arrangement were similar to those in our previous report,^[^ ^10^ ^]^ in which uniform MCTSs were formed by an additional washing step of cells not lodged in the microwells 1 h after cell seeding. Conversely, by overlapping the recasting zone between microwells, the microwell array could be greatly compacted to minimize unwanted cell seeding in dense arrangement. Huh‐7 MCTSs with a fixed initial seeding density of 2 × 10^4^ cells per well were seeded onto sparse and dense microwell arrays with washing step involved. MCTSs formed in a sparse microwell array after 4 days were shown to be irregular in size and morphology, while MCTSs harvested from a dense microwell array had a uniform shape and size with good viability, as presented in both differential interference contrast (DIC) and fluorescent (live/dead staining) images (Figure 3B). MCTSs retrieved from the dense microwell array were more focused and narrower with regards to the size distribution than those retrieved from the sparse array (Figure 3C). The average diameters of the MCTS showed no significant difference between the two conditions; however, the standard deviation in the size of the MCTSs formed in the dense group was approximately half than that of the sparse group (Figure 3D and Table S4, Supporting Information). The results indicated that the improvement of the microwell array from sparse to dense design not only reduced the additional step required and unwanted cell loss but also directly increased the uniformity of the MCTS.

Emphasis was placed on the microwell array arrangement to reduce the nonutilized surface area upon cell seeding (Figure 3). A few previous works have proposed various structures and array arrangements with decreased cell loss during cell seeding for cell spheroid formation.^[^ ^5^, ^6^, ^8^, ^31^ ^]^ In short, the desired microwell array should be as compact as possible while maintaining the marginal thickness of the barrier between microwells to enable surface utilization and compartmentalization. Although the recast region of the microwell was sought to be a disadvantageous byproduct due to the laser thermal‐heating process, the empirical examination showed that the wing recast structures could be properly overlapped, forming a thin and wall‐like structure by optimizing the horizontal *d*_x_ and vertical *d*_y_ of the microwell array (Figure 3A). The results demonstrated that the improved microwell array arrangement could not only yield MCTSs with a much improved homogeneous size distribution (Figure 3B,C) but also minimize cell loss, as no additional washing step was involved after cell seeding. From the perspective of cost and labor, the current approach provides direct “writing” without the need for a cleanroom, expensive equipment, unusual materials, or laborious protocols.

**Figure 4:**
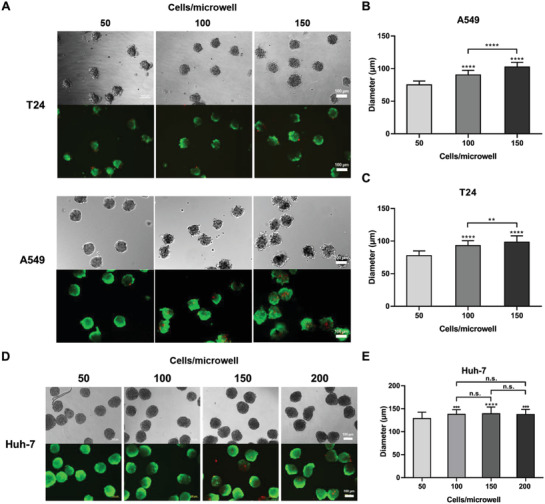
Comparison of Huh‐7, A549, and T24 MCTSs at different seeding densities. A) DIC and fluorescent (live/dead staining) images of A549 and T24 MCTSs with various initial seeding densities (1.65 × 10^4^, 3.3 × 10^4^, and 4.95 × 10^4^ cells per well). Quantifications of the B) A549 and C) T24 MCTS diameter with respect to the initial seeding density (*n* = 50). D) DIC and fluorescent (live/dead staining) images of Huh‐7 MCTSs with various initial seeding densities (1.65 × 10^4^, 3.3 × 10^4^, 4.95 × 10^4^, and 6.6 × 10^4^ cells per well). E) Quantification of the Huh‐7 MCTS diameter with respect to the initial seeding density (*n* = 50). Scale bar = 100 µm.

The designed dense microwell arrangement was further validated on three types of cancer cells A549, T24, and Huh‐7 in the formation of MCTSs over three different seeding densities of 50, 100, and 150 cells/microwell (**Figure** **4**). The table showing the corresponding seeding density of cells per well of the 96‐well plate was also appended (Table S5, Supporting Information). DIC and live/dead staining images indicated that A549, T24, and Huh‐7 MCTSs formed with uniform size and good viability (Figure 4A,D). While A549 and T24 MCTS diameters increased steadily from ≈75 to 100 µm with densities from 50 to 150 cells/microwell (Figure 4B,C), Huh‐7 cells grew MCTSs with diameters of ≈128 µm at 50 cells/microwell and showed no significant increase in size at ≈139 µm at both 100 and 150 cells/microwell (Figure 4D,E). To examine whether a plateau of growth was reached in Huh‐7 MCTSs, an additional seeding density at 200 cells/microwell was evaluated, and it was found that MCTSs displayed a cap of the average diameter at ≈138 µm under the designed microwell parameters (Table S6, Supporting Information).

Understanding of the confinement of MCTS growth under a particular microwell parameter offers a foundation for system characterization and prospective protocol optimization (Figure 4). It was noted that T24 and A549 formed MCTS with similar size ranges under the same seeding density, while Huh‐7 grew to a larger MCTS. This outcome may be attributed to a few factors, such as the size of individual cells, the proliferation over the period of culture, and the compactness of each MCTS. Interestingly, the plateau in the size of Huh‐7 MCTSs with different cell seeding densities may imply a capping effect after 4 days of formation, likely caused by the physical constraints imposed by the microwells to limit the size of MCTSs. On the one hand, it can be observed that the Huh‐7 MCTSs appeared light gray at 50 cells/microwell and grew darker as the cell densities increased, suggesting denser and more compact MCTSs formed at 200 cells/microwell (Figure 4D). On the other hand, upon increasing the microwell size to 470 µm, ≈100 cells/microwell of Huh‐7 MCTSs could grow to ≈220 µm after 4 days (data not shown). These results highlight the important roles of the designed dimensions of the microwells and number of seeded cells from different cell lines in regulating the size of MCTSs.

### 2.3 2D and 3D Multicellular Tumor Spheroid Anticancer Drug Screening

**Figure 5:**
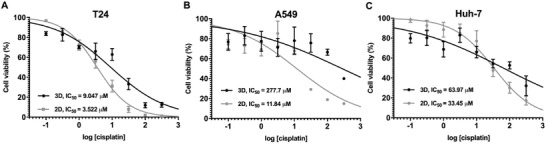
Dose‐response analysis of the cisplatin treatment on A) T24, B) A549, and C) Huh‐7 in both MCTS (3D) and 2D conditions (*n* = 3).

Cisplatin is a well‐known drug approved by the Food and Drug Administration for the treatment of numerous human cancers, including lung, bladder, and ovarian cancers.^[^ ^32^ ^]^ To assess the effect of cisplatin treatment on the three types of cancers, dose‐response assays were applied to evaluate the viability of both 2D and 3D MCTS cultures (**Figure** **5** and Table S7, Supporting Information). Based on the aforementioned results of the MCTS diameter generated in different seeding concentration, T24 and A549 were selected at ≈100 µm (150 cells/microwell) and Huh‐7 at ≈128 µm (50 cells/microwell) that showed similar range in sizes. The results displayed that while all cancers showed a dose‐dependent response in both 2D and 3D conditions at a range of concentrations from 0 to 320 µM, notable differences were discovered with regard to the drug resistance to cisplatin between different cancer types and culture conditions. T24 showed similar drug sensitivity in both 2D and 3D with IC_50_ values of 3.52 and 9.05 µM, respectively (Figure 5A). Contrarily, the IC_50_ of A549 displayed more drug resistance in the 3D condition with a value of 277.7 µM than that in the 2D condition with a value of 11.84 µM (Figure 5B). Although Huh‐7 revealed similar IC_50_ values of 33.45 and 63.97 µM in both 2D and 3D conditions, the 2D condition was found to be more drug resistant at low doses than that of the 3D condition (Figure 5C). Among these results, A549 MCTS generated from the microwells differed significantly from T24 and Huh‐7 in terms of their drug sensitivity toward different lineage and culture conditions. Given that matrix distribution and compactness can be modulated by the 3D structure of MCTS, we wondered these differences could be associated with the effectiveness of drug penetration.

### 2.4 Diffusional Permeability Coefficients of Multicellular Tumor Spheroids

**Figure 6:**
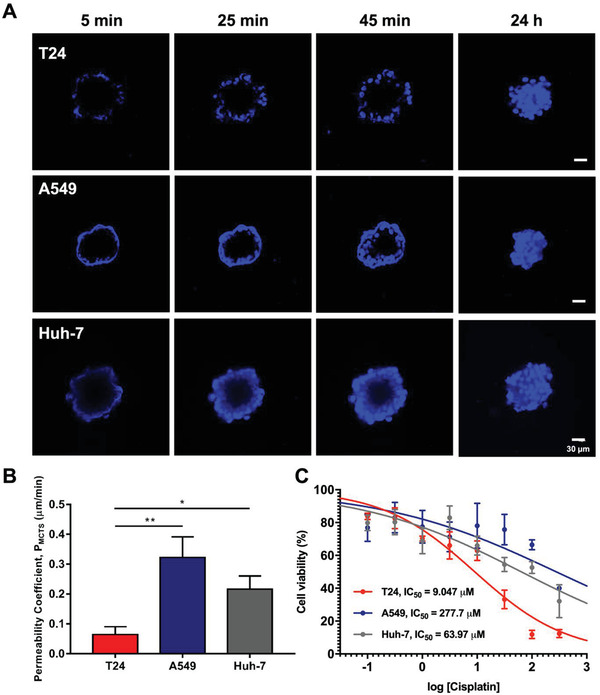
Quantification of the permeability coefficient (*P*_MCTS_), time‐lapse confocal images and the effect of increasing cisplatin concentration on T24, A549, and Huh‐7 MCTSs. A) Time‐lapse Hoechst fluorescent images of MCTSs. B) Permeability coefficients (*P*_MCTS_) of all MCTSs. C) Dose‐response curves of T24, A549, and Huh‐7 cell viability under MCTS conditions. All error bars represent the standard deviation of the measurement (*n* = 3). Scale bar = 30 µm.

Therefore, to understand the dynamics of drug permeability into the T24, A549 and Huh‐7 MCTSs, Hoechst staining was applied to mimic the diffusion of small‐molecule anticancer drugs through all three types of MCTSs (**Figure** **6**). Hoechst was chosen not only for its similarity in molecular weight (452.56 Da) but also because the site of action of cisplatin was through interacting with nucleus DNA to induce DNA adducts.^[^ ^33^ ^]^ Diffusion of the Hoechst dye could be observed through the MCTSs after staining for 5, 25, and 45 min, respectively (Figure 5A). The stain was diffused thoroughly into the middle part of the three types of MCTSs after 24 h of incubation that served as the control. The *P*_MCTS_ values were measured for all three types of MCTS, where the *P*_MCTS_ of T24 was the lowest at ≈0.067 µm min^−1^, compared to A549 at 0.322 µm min^−1^ and Huh‐7 at 0.217 µm min^−1^ (Figure 5B and Table S7, Supporting Information). Previous studies reported that doxorubicin penetration through MCF‐7 breast MCTSs was ≈0.16 µm min^−1^,^[^ ^19^ ^]^ which is comparable to the *P*_MCTS_ of the group in our study, and the efficient permeability of Caco‐2 MCTSs ranged from 0.3 to 1.2 µm min^−1^.^[^ ^18^ ^]^

In the present study, one of the crucial goals was not only to optimize the consistency and arrangement of the microwell array fabrication but also to validate that the microwell platform that was conducive for both growing MCTSs originating from different cancer types and integrating them into a conventional screening of chemotherapeutic agent workflow. In addition, we desired to identify the effects of culturing conditions in either 2D monolayers or 3D MCTSs to the susceptibility of the drug. For this purpose, three cell lines originating from bladder, lung, and liver cancers were assessed for evaluating their susceptibility to cisplatin (Figure 5). The results indicated that both the cancer type origin and the culturing conditions played crucial roles in determining the susceptibility of the drug. For example, in agreement with the clinical utility of cisplatin treatment for bladder cancer,^[^ ^32^ ^]^ the T24 cells showed similar IC_50_ values for cisplatin regardless of the culturing conditions in both 2D and MCTSs. Contrarily, the 3D MCTSs of the A549 and Huh‐7 cells displayed a greater cisplatin resistance that of the 2D monolayer culture, implying that changing the condition to MCTSs substantially promoted the resistance to cisplatin in these two cells lines. Indeed, similar findings were reported in the previous studies that the resemblance of 3D A549 and Huh‐7 spheroids to tissue structures increased the resistance to anticancer drugs.^[^ ^34^, ^35^ ^]^

Given that the structure formed in MCTS may serve as a physical barrier to prevent drug penetration, we then further looked into whether the drug efficacy was mediated by interstitial transport in different cancer types (Figure 6). The results indicated that the similar size of MCTSs originated from that different cancer types inherently possessed different levels of permeability. Jena et al. also reported a similar finding on the comparison of murine liver and human breast MCTSs, in which the permeability could differ considerably with the different endogenous ECM distribution and interstitial spacing over different tissue types.^[^ ^36^ ^]^ In our results, the T24 MCTSs showed the lowest *P*_MCTS_ (0.052 µm min^−1^) but were the most sensitive to cisplatin treatment (IC_50_ = 9.0 µM), as opposed to the most resistive A549 MCTSs (IC_50_ = 277.7 µM) that exhibited approximately three times higher *P*_MCTS_ (0.322 µm min^−1^). Considering the 24‐h experimental design in this study, if the 2D monolayer culture was directly exposed to the cisplatin upon drug incubation, the delay of cisplatin to thoroughly diffuse to the core of the 3D MCTSs was 10 h at most based on the *P*_MCTS_ measured. The exact diffusion of cisplatin, rather than the use of Hoechst staining in the permeability measurement, was expected to take place in an even faster manner due to the drug‐induced apoptosis, indicating that a sufficient amount of time for drug interaction was established prior to the cytotoxicity test.

Therefore, we attributed that the permeability in our current avascular MCTS model might have less of an effect on the drug efficacy than the culturing conditions and tissue origins did. To verify these claims, we also investigated the Huh‐7 MCTSs, with the *P*_MCTS_ values showing significant differences at two initial cell seeding densities of 50 and 200 cells/microwell, with values of ≈0.138 and 0.046 µm min^−1^ (Figure S2, Supporting Information), whereas the IC_50_ of cisplatin was almost identical. This result further highlighted that if the cell and matrix compactness affect drug permeability only in a short period of time, such as the proposed avascular MCTS model (≈100 µm in diameter), the mechanism of action of cisplatin in the individual cancer as well as the pronounced cell‐cell interactions through 3D structures may play an even more crucial role in the corresponding drug sensitivity.^[^ ^37^ ^]^

## 3 Conclusions

In summary, we developed a micro U‐well platform featuring a highly reliable fabrication with an optimally arranged microwell array. The proposed configuration of the microwell array could be achieved by a simple and rapid CO_2_ laser microfabrication method and produced uniform and size‐controlled MCTS. MCTSs were used as an in vitro tumor‐mimicking model to probe tumor permeability and served as an effective tool for the discovery of therapeutic drug screening for cancer treatment. The current approach is envisioned as an in‐depth protocol guide for a facile and highly consistent microwell fabrication to reliably produce MCTSs and establish a workflow for drug evaluation.

## 4 Experimental Section

##### Microwell Fabrication and Characterization

**Figure 7:**
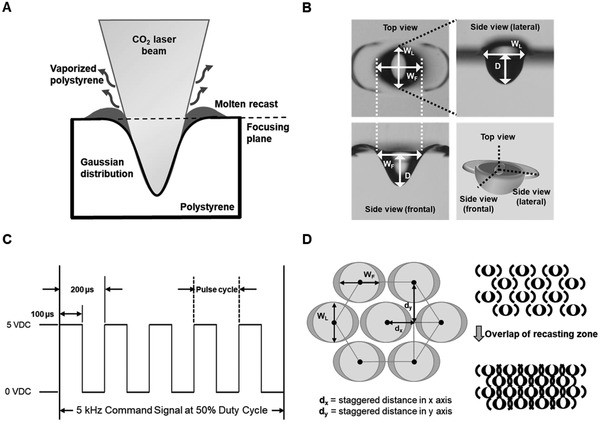
Illustration of the CO_2_ laser ablation process, the definition of the microwell geometry and command signals. A) Gaussian profile of a CO_2_ laser beam used to ablate the surface of polystyrene to create a concave hole with a remnant of molten recast. B) Top, side‐frontal, and side‐lateral views of microwells. C) Laser pulse command for microwell fabrication. D) Control over the hexagonal packing of microwells, staggered distance on the x‐ and y‐axis between microwells; *d*_x_ and *d*_y_, are two spacing parameters. Microwell arrays can be “overpacked,” resulting in overlap of the recasting zone.

Polystyrene (PS) slides were utilized as the substrate for the fabrication and analysis of microwells (**Figure** **7**). A 10 W CO_2_ laser ablation system with a 10.6 µm wavelength was operated to perform single‐dot ablation, with motorized x‐y stages to position the substrate at the location of interest. These above parts were assembled by Laser Solution Technology Inc., Taipei, Taiwan based on the author's conceptual design. Ablated PS presented a profile of Gaussian distribution due to heat melt and vaporization (Figure 7A), which resulted in a concave micro U‐well structure with recasts residing horizontally at the edge. Given that the laser beam was focused by a 2'’ lens, the size of the microwell was in the approximate range of 100–500 µm.

Microwells were characterized according to the geometric orientation after ablation (Figure 7B). After the fabrication of the microwell, surface debris was removed with an air spray gun. The side view of the microwell was acquired by replica molding with PDMS. The top view revealed a slightly elliptical concave well formation with two dark protruding structures, defined as wing structures, along the horizontal axis. The asymmetric difference in elliptical shape was defined in terms of the side‐frontal and side‐lateral views. The laser command signal was operated by pulse width modulation, and the parameters were investigated in the following ranges: frequency from 1 to 20 kHz, duty cycle from 10% to 60% and pulse number from 100 to 600. For example, a series of laser pulses at 5 kHz and a 50% duty cycle corresponded to each laser pulse being on for 100 μs and off for 100 μs during a 200 μs interval (Figure 7C). The number of pulses was regulated by the number of pulse cycles during each single‐dot ablation process. By adjusting these parameters, a variety of command signals were delivered to fabricate different microwell profiles. To avoid unwanted space between microwells, a microwell array was designed in a staggered arrangement (Figure 7D); d_x_ and d_y_ were spacing parameters that could be empirically determined through overlapping the recasting zone. The TW performed on each microwell ablation could be calculated by the following equation:

(1) $$\begin{array}{c} \text{TW}\;\left(J\right)=10\;\left(W\right)\times \frac{1}{\text{Frequency}}\;\left(s\right)\times \text{Duty}\;\text{cycle}\;\left(\%\right)\times \text{Number}\;\text{of}\;\text{pulses} \end{array}$$

##### Scanning Electron Microscopy

The microwell samples were cut into appropriate sizes and rinsed several times with deionized water and ethanol. The samples were air‐dried and then treated with vacuum‐sputtered platinum (JEOL JFC‐1600; JEOL Ltd., Tokyo, Japan). The surfaces of the microwell structures were then observed by SEM (JSM‐7001F; JEOL Ltd.).

##### Cell Culture

Huh‐7, a human hepatocarcinoma cell line, and A549, an adenocarcinomic human alveolar basal epithelial cell line, were both cultured in 10 cm dishes (Nunclon; Thermo Fisher Scientific, Waltham, MA, USA) and maintained in high‐glucose Dulbecco's modified Eagle medium supplemented with 10% fetal bovine serum (Gibco, Gaithersburg, MD, USA) and 1% penicillin streptomycin solution (Corning, NY, USA). T24, a human bladder carcinoma cell line, was cultured in 10 cm dishes and maintained in McCoy's 5a medium (modified) with l‐glutamine (1.5 mm) adjusted to contain sodium bicarbonate (2.2 g L^−1^) and supplemented with 10% fetal bovine serum (Gibco) and 1% penicillin streptomycin solution (Corning). The cells were kept at 37 °C in a 5% CO_2_ humidified chamber. The media were monitored daily and replaced regularly every 2 to 3 days. The cells were subcultured using 0.5% trypsin‐EDTA (Corning) upon reaching 80–90% confluence.

##### Formation of Multicellular Tumor Spheroids

T24, A549, and Huh‐7 MCTSs were generated by forced aggregation of the cells in microwells fabricated via CO_2_ laser ablation on a 96‐well plate, with each well containing 330 microwells (Figure S3, Supporting Information). Before cell seeding, the microwells were incubated with 75% ethanol to remove debris, and then ultraviolet light (15 W) was applied to irradiate the surface of the microwells for 1 h for sterilization. To prevent undesired cell attachment to the microwell plates, microwells were immersed in 0.2% Pluronic F127 (Sigma‐Aldrich Corp., St. Louis, MO, USA) in 1X PBS for at least 40 min, followed by two 1X PBS rinses. The desired range of cell densities was determined, and cell suspension (200 µL) was seeded in each well and kept for 4 days. MCTSs could be easily retrieved by gentle pipetting with serum‐free medium to flush them out from the microwells.

##### Multicellular Tumor Spheroid Viability Assessment

A LIVE/DEAD viability assay kit (Invitrogen, Carlsbad, CA, USA) was used to assess cell viability. Calcein‐AM staining solution (1:2000 dilution) and EthD‐1 staining solution (1:1000 dilution) were added and incubated at 5% CO_2_ and 37 °C for at least 1 h prior to image capture. To avoid breakage or dislodging of cells from MCTS during the pipetting steps, all MCTS were kept in the microwells throughout the staining procedure and were only carefully harvested prior to the optical and fluorescent observation.

##### Imaging and Quantification

All images were taken with either an Olympus IX83 or CKX53 inverted microscope (Olympus, Tokyo, Japan) and analyzed by ImageJ. Fluorescence images of the permeability study were obtained using a FV‐1000 confocal laser scanning microscope (Olympus). The permeability was quantified by the mean fluorescence of the MCTS.

##### Drug Screening

A549, T24, and Huh‐7 cells were cultured in conventional 96‐well plates (100 µL well^−1^) at a concentration of 5 × 10^4^ cells mL^−1^. After 1 day, cisplatin stock solutions were serially diluted in growth medium to final concentrations of 0.1, 0.32, 1, 3.2, 10, 32, 100, and 320 µM. The growth medium was then replaced with diluted cisplatin solution (100 µL) and incubated for another 24 h. Prior to imaging, Cell Counting Kit‐8 (CCK‐8) assay solution (10 µL well^−1^) was added to the wells (110 µL) to assess cell viability under cisplatin treatment. Then, the solution (80 µL) was transferred to an opaque 96‐well plate. The different drug concentrations were tested in quadruplicate, and the negative control (cell free) was used to determine any background fluorescence. A microplate reader (EMax Plus; FortéBio, Fremont, CA, USA) was utilized to measure the absorbance with a 450 nm filter. Because the absorbance of CCK‐8 is directly proportional to cell viability, the average cell viability for each drug concentration was calculated by normalizing each value to the untreated control at 0 µM, and the half maximal inhibitory concentration (IC_50_) was determined using Prism 7.0 (GraphPad Software, San Diego, CA, USA).

To perform drug screening on MCTSs (≈110 μm in diameter), A549 and T24 cells were seeded with the cell suspension (200 µL well^−1^) at a concentration of 2.5 × 10^5^ cells mL^−1^, and Huh‐7 cells were seeded with the cell suspension (200 µL well^−1^) at concentration of 8.25 × 10^4^ cells mL^−1^ in microwells fabricated on the untreated 96‐well plate with the desired combination of laser parameters. Cisplatin stock solutions were serially diluted in growth medium to 2X desired concentrations of 0.2, 0.64, 2, 6.4, 20, 64, 200, and 640 µM. On day 4, the supernatant (100 out of 200 µL) was removed from the wells, and the diluted cisplatin solution (100 µL) was carefully added and mixed with the remaining medium (100 µL) to reach the desired concentration and incubated for 1 day. Then, CCK‐8 assay solution (20 µL well^−1^) was added to the wells (220 µL) to assess cell viability after cisplatin treatment. Then, the solution (80 µL) was transferred to an opaque 96‐well plate. The remaining steps were the same as in 2D drug screening to determine the IC_50_.

##### Permeability Measurement

The MCTSs were transferred to a laboratory‐made chamber slide in suspension (100 µL per chamber) for ease of observation under a laser confocal microscope. Then, Hoechst nucleic acid staining solution (100 µL; 1:1000 dilution; 33 258, Invitrogen) diluted in PBS was gently added to the chamber with minimal pipetting interference to ensure proper diffusion of the dye. Given that the confocal laser was limited to an ≈60 µm thick section of the MCTSs, the z‐stack images (4 µm per stack) of the MCTSs were recorded from 0 to 60 µm, which was considered the middle section of the MCTSs. The diffusion of the Hoechst into the MCTSs was imaged at 5, 25, and 45 min.

The average intensity values of each MCTS were extracted to calculate the *P*_MCTS_, as shown in Equation (2). The equation was derived and modified from Fick's first law^[^ ^38^ ^]^ and a previous study,^[^ ^39^ ^]^ where *P*_MCTS_ was defined as the following equation:

(2) $$\begin{array}{c} P_{\text{MCTS}}=\frac{F_{H}}{A_{\text{MCTS}}\Delta C} \end{array}$$

Here, *F*_H_ refers to the diffusion flux of dye across the MCTS; *A*_MCTS_, the surface area of the MCTS where diffusion occurs; and Δ*C*, the concentration difference of the diffusing solute across the MCTS. From mass conservation, *F*_H_ can be defined as the rate of change in the diffusion of Hoechst, H, within the MCTSs described using the following equation:

(3) $$\begin{array}{c} F_{H}=\frac{dH}{dt_{\text{MCTS}}}=\frac{d}{dt}\underset{\text{MCTS}}{\int }Cd\forall \end{array}$$

where *C* denotes the local dye concentration of Hoechst per u nit volume, and *d∀*, an infinitesimal volume within the MCTS. Considering *C* to be directly proportional to its fluorescence intensity, *I*, and substituting Equation (3) into Equation (2), the relation is obtained in the following equation:

(4) $$\begin{array}{c} P_{\text{MCTS}}=\frac{\frac{d}{dt}\int_{\text{MCTS}}Id\forall }{A_{\text{MCTS}}\cdot \Delta I} \end{array}$$

Given that:

(5) $$\begin{array}{c} I_{\text{ave},\;\;\text{MCTS}}=\frac{1}{\forall_{\text{MCTS}}}\underset{\text{MCTS}}{\int }Id\forall \end{array}$$

Equation (4) becomes:

(6) $$\begin{array}{c} P_{\text{MCTS}}=\frac{\forall_{\text{MCTS}}\frac{d}{dt}I_{\text{ave},\;\text{MCTS}}}{A_{\text{MCTS}}\Delta I} \end{array}$$

As we assumed the MCTSs to be spheres, $V_{\text{MCTS}}=\frac{4}{3}\pi r^{3}$, *A*_MCTS_ = *πr* ^2^, Equation (6) becomes:

(7) $$\begin{array}{c} P_{\text{MCTS}}=\frac{\frac{4}{3}\pi r^{3}\frac{d}{dt}I_{\text{ave},\;\text{MCTS}}}{\pi r^{2}\Delta I}=\frac{r}{3}\frac{I_{\text{ave}|t}-I_{\text{ave}|t\;=\;5}}{I_{\text{ave}|\text{control}}-I_{\text{ave}|t\;=\;5}} \end{array}$$

where *r* refers to the mean radius of the MCTS; I_ave_|_*t* = 5_ and *I*_ave_|_*t*_ are the average fluorescence intensity at time *t* = 5 min and each recorded time *t*, respectively; and *I*_aver|control_ is the average intensity of fluorescence within the MCTS area after 24 h of incubation.

##### Statistical Analysis

All data in the study are presented as the mean ± standard deviation. For the statistical analysis, statistical significance was determined via one‐way analysis of variance with Prism 7.0 (GraphPad Software). A *p*‐value < 0.05 was considered significant.

## Conflict of Interest

The authors declare no conflict of interest.

## Supporting information

Supporting Information
