# Supplementary material for: A Highly Reproducible Micro U‐Well Array Plate Facilitating High‐Throughput Tumor Spheroid Culture and Drug Assessment
Source: Glob Chall. 2020 Nov 4;5(2):2000056. doi: 10.1002/gch2.202000056 (PMC7857131; doi:10.1002/gch2.202000056)
Supplement: Supplementary file 1 — Supporting Information [file GCH2-5-2000056-s001.pdf]

© 2020 Wiley-VCH GmbH

Global Challenges
Open Access

Supporting Information

for *Global Challenges*, DOI: 10.1002/gch2.202000056

A Highly Reproducible Micro U-Well Array Plate Facilitating High-Throughput Tumor Spheroid Culture and Drug Assessment

*Kuang-Wei Wu, Ching-Te Kuo, and Ting-Yuan Tu**

WILEY-VCH

© 2020 Wiley-VCH GmbH

Supporting Information

A highly reproducible micro U-well array plate facilitating high-throughput tumor spheroid culture and drug assessment

Kuang-Wei Wu[1], *Prof. Ching-Te Kuo*[2], *Prof. Ting-Yuan Tu* [134*]

[1]Department of Biomedical Engineering, National Cheng Kung University, Tainan 70101, Taiwan
[2]Department of Mechanical and Electro-Mechanical Engineering, National Sun Yat-sen University, Kaohsiung 80400, Taiwan
[3]Medical Device Innovation Center, National Cheng Kung University, Tainan 70101, Taiwan
[4]International Center for Wound Repair and Regeneration, National Cheng Kung University, Tainan 70101, Taiwan

* To whom correspondence should be addressed (tingyuan@mail.ncku.edu.tw)

Keywords: rapid prototyping, CO_2 laser, multicellular tumor spheroids, in vitro tumor models, microwells, permeability coefficient, cisplatin

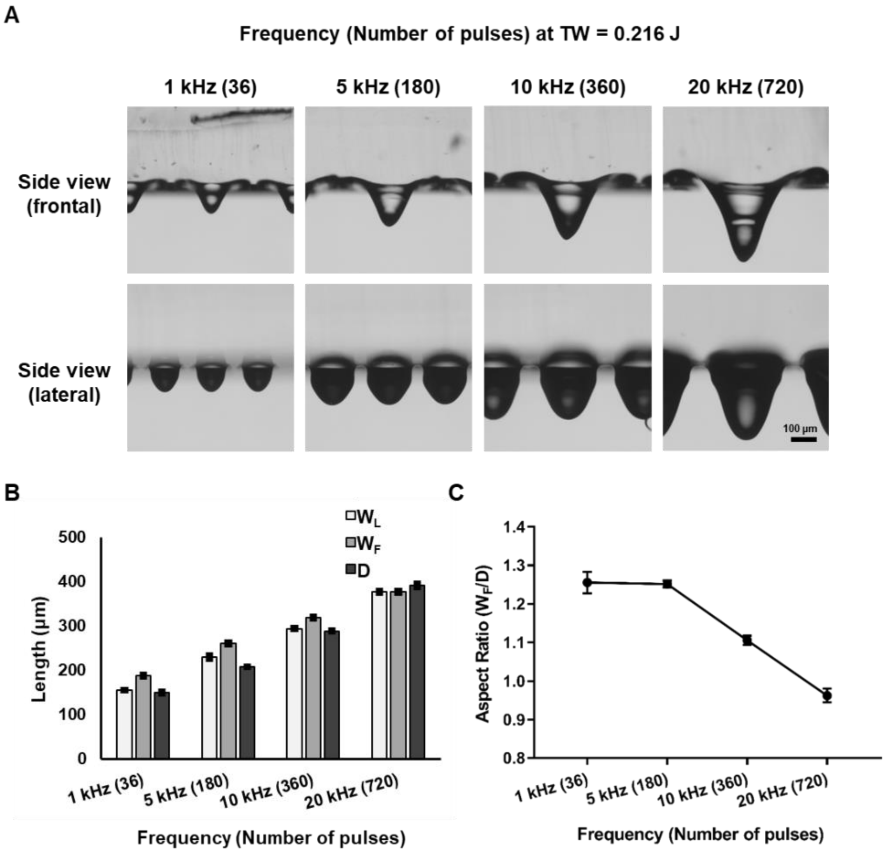

Figure S1. Microwells fabricated via different frequencies under fixed TW. (A) Frontal and lateral side views of microwells at different frequencies. (B) Characterization of microwells at different frequencies. (C) Aspect ratio of microwells.

Table S1. Microwell dimensions at different number of pulses.

Average of Frontal width, Depth, and Aspect ratio = W_F/D (μm) ± standard deviation						
**Number of pulses**	**60**	**120**	**180**	**300**	**500**	**700**
**Width frontal**	206.6 ± 2.11	256.0 ± 2.61	262.4 ± 4.53	288.0 ± 3.20	332.8 ± 1.60	366.9 ± 3.70
**Depth**	160.0 ± 2.61	190.4 ± 1.85	211.2 ± 2.61	254.4 ± 4.23	327.5 ± 2.85	383.5 ± 0.92
**AR value**	1.3 ± 0.01	1.3 ± 0.02	1.2 ± 0.01	1.1 ± 0.02	1.0 ± 0.01	1.0 ± 0.01

Table S2. Microwell dimensions with combinations of duty cycle and number of pulses.

Average of Width and Depth (μm) ± standard deviation			
**Duty cycle (Number of pulses)**	**15% (120)**	**30% (60)**	**60% (30)**
**Width frontal**	124.8 ± 2.26	139.3 ± 1.60	157.5 ± 3.56
**Width lateral**	104.8 ± 2.07	120.3 ± 3.42	138.9 ± 3.42
**Depth**	98. 7 ± 2.04	116.4 ± 2.01	134.40 ± 2.61
**AR value**	1.3 ± 0.04	1.2 ± 0.03	1.2 ± 0.03

Table S3. Microwell dimensions under different frequency at same total work.

Average of Width and Depth (μm) ± standard deviation				
Frequency (Number of pulses)	1 kHz (36)	5 kHz (180)	10 kHz (360)	20 kHz (720)
Width frontal	188.8 ± 2.61	261.1 ± 2.94	319.3 ± 3.24	377.6 ± 2.61
Width lateral	155.2 ± 1.31	230.1 ± 3.71	294.4 ± 1.74	376.8 ± 3.06
Depth	150.4 ± 2.61	208.5 ± 1.26	288.8 ± 1.51	392.4 ± 4.57
AR value	1.3 ± 0.03	1.3 ± 0.01	1.4 ± 0.03	1.2 ± 0.02

Table S4. MCTS diameter with respect to sparse and dense arrangement of microwell array

Average of MCTS diameter (μm) ± standard deviation	
Arrangement	2 × 10^4 cells/well
Sparse	132.5 ± 29.70
Dense	137.0 ± 11.31

Table **S5.** Conversion of cell seeding density.

330 microwell / well (96 well plate)	
Cells / well	Cells / microwell
1.65E+4	50
3.3E+4	100
4.95E+4	150
6.6E+4	200

Table S4. Comparison of T24, A549 and Huh-7 MCTS diameter at different seeding densities.

	Average of MCTS diameter (μm) ± standard deviation			
Cell type	50 cells / microwell	100 cells / microwell	150 cells / microwell	200 cells / microwell
T24	77.6 ± 7.33	93.1 ± 7.56	98.4 ± 9.65	-
A549	75.0 ± 5.88	90.2 ± 7.12	102.4 ± 7.36	-
Huh-7	128.6 ± 14.03	137.2 ± 10.10	139.1 ± 14.63	137.6 ± 10.99

Table S5. Diameter, IC_{50} (μM) assayed for cisplatin resistance, and permeability coefficient (P_{MCTS}) in 2D and 3D MCTS conditions.

Cell seeding density (Huh-7: 1.65 × 10^4 cells/well, A549, T24: 4.95 × 10^4 cells/well)						
Cell type	T24		A549		Huh-7	
Condition	2D	3D MCTS	2D	3D MCTS	2D	3D MCTS
Diameter (μm)	-	98.4	-	102.4	-	128.6
IC_{50} (μM)	3.52	9.05	11.84	277.70	33.45	63.97
P_d (μm/min)	-	0.067	-	0.322	-	0.217

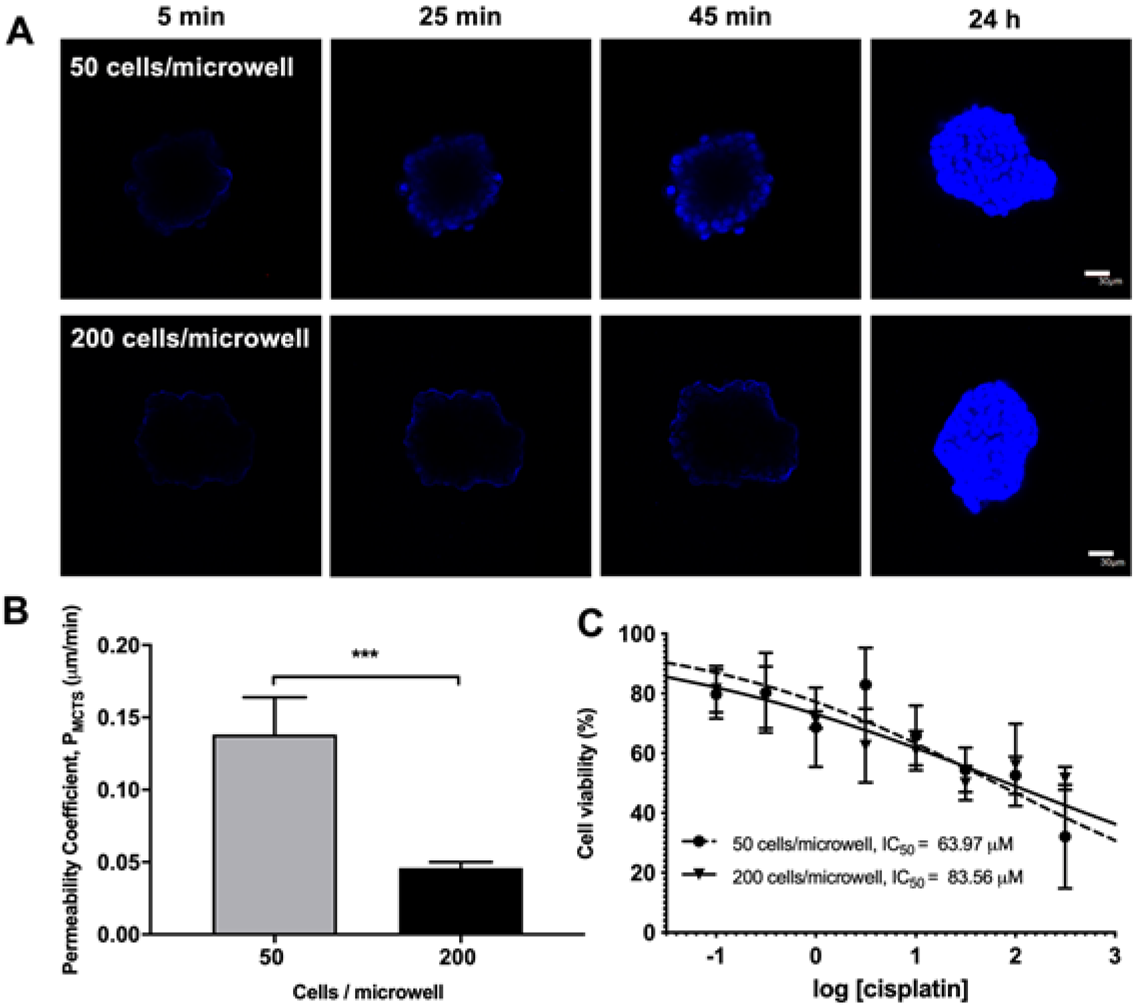

Figure S2. Quantification and time-lapse confocal images of MCTS at 50 and 200 cells/microwell seeding density. (A) Time-lapse Hoechst fluorescent image of MCTS at the 60-µm section position. (B) Permeability coefficient (PMCTS) at 50 and 200 cells/microwell seeding density. (C) Dose-response curves of Huh-7 MCTS conditions at 50 and , A549, and T24 cell viability under MCTS at 50 and 200 cells/microwell seeding density.

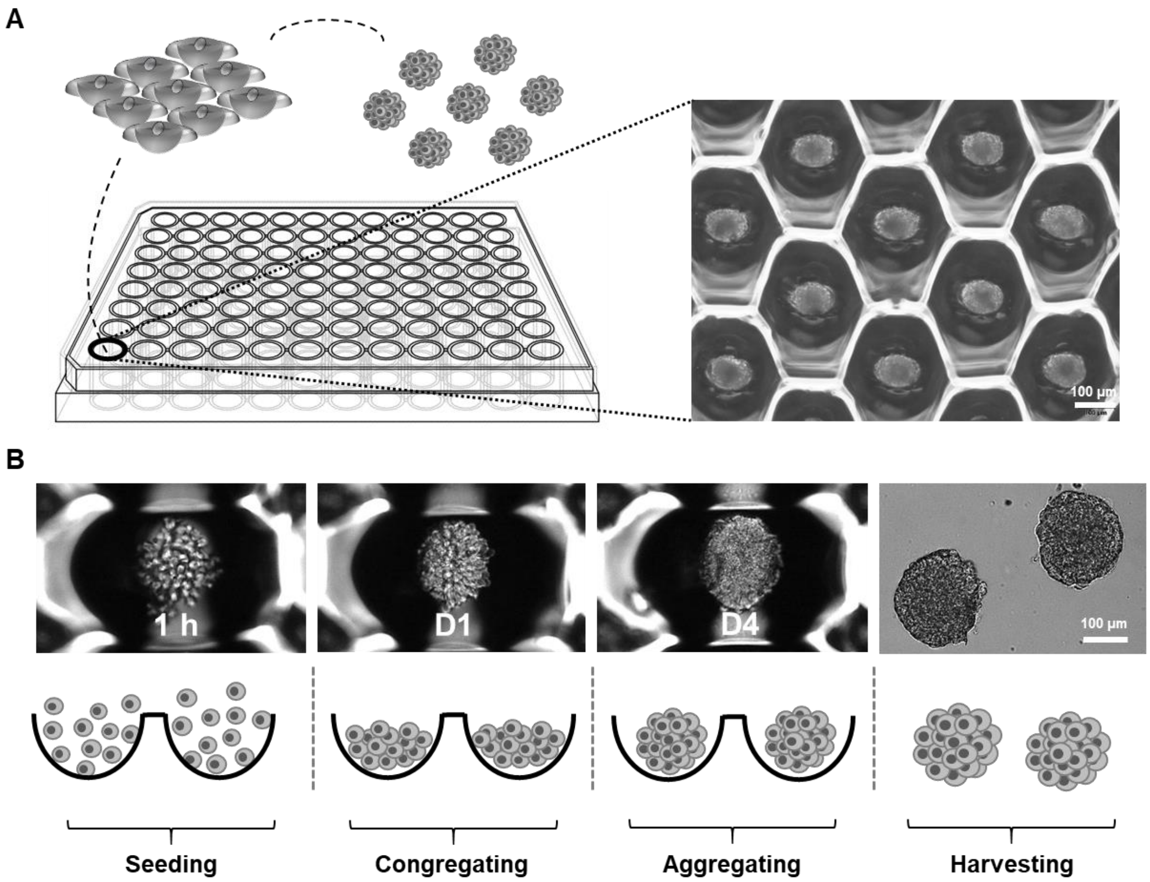

Figure S3. Schematic diagram of the staggered arrangement of microwells and the overlap of the recasting zone. (A) MCTSs formed in microwells fabricated via CO_2 *laser ablation on a 96-well plate. (B) Morphology of Huh-7 MCTS cultured for 1 hour, 1 day, and 4 days and after harvesting.*
